# Randomised, Placebo-Controlled Investigation of the Impact of Probiotic Consumption on Gut Microbiota Diversity and the Faecal Metabolome in Seniors

**DOI:** 10.3390/microorganisms12040796

**Published:** 2024-04-15

**Authors:** Gabriella C. van Zanten, Anne Lundager Madsen, Christian C. Yde, Lukasz Krych, Nicolas Yeung, Markku T. Saarinen, Witold Kot, Henrik Max Jensen, Morten A. Rasmussen, Arthur C. Ouwehand, Dennis S. Nielsen

**Affiliations:** 1 Department of Food Science, University of Copenhagen, 1958 Frederiksberg, Denmark; gabivanzanten@gmail.com (G.C.v.Z.); annelmadsen@sund.ku.dk (A.L.M.); krych@food.ku.dk (L.K.); mortenr@food.ku.dk (M.A.R.); dn@food.ku.dk (D.S.N.); 2 IFF Enabling Technologies, Brabrand, 8220 Aarhus, Denmark; christian-clement.yde@iff.com (C.C.Y.); henrik.max.jensen@iff.com (H.M.J.); 3 IFF Health, 02460 Kantvik, Finland; nicolas.yeung@iff.com (N.Y.); markku.saarinen@iff.com (M.T.S.); 4 Department of Plant and Environmental Sciences, University of Copenhagen, 1871 Frederiksberg, Denmark; wk@plen.ku.dk; 5 Copenhagen Studies on Asthma in Childhood, University of Copenhagen, 2820 Gentofte, Denmark

**Keywords:** *Lactobacillus*, *Bifidobacterium*, probiotic, older adults, faecal microbiota, faecal microbial metabolites

## Abstract

Aging has been associated with a changed composition and function of the gut microbiota (GM). Here, we investigate the effects of the multi-strain probiotic HOWARU^®^ Restore on GM composition and function in seniors. Ninety-eight healthy adult volunteers aged ≥75 years were enrolled in a randomised, double-blinded intervention (NCT02207140), where they received HOWARU Restore (10^10^ CFU) or the placebo daily for 24 weeks, with 45 volunteers from each group completing the intervention. Questionnaires monitoring the effects on gastro-intestinal discomfort and bowel movements were collected. Faecal samples for GM characterisation (qPCR, 16S rRNA gene amplicon sequencing) and metabolomics (GC-FID, ^1^H NMR) were collected at the baseline and after 24 weeks. In the probiotic group, self-reported gastro-intestinal discomfort in the form of flatulence was significantly decreased during the intervention. At the baseline, 151 ‘core species’ (present in ≥95% of samples) were identified. Most core species belonged to the *Lachnospiraceae* and *Ruminococcaceae* families. Neither alpha diversity nor beta diversity or faecal metabolites was affected by probiotic intake. On the contrary, we observed high intra-individual GM stability, with ‘individual’ accounting for 72–75% of variation. In conclusion, 24 weeks of HOWARU Restore intake reduced gastro-intestinal discomfort in the form of flatulence in healthy seniors without significantly influencing GM composition or activity.

## 1. Introduction

With biological aging, the ability of cells and tissues in the body to repair and regenerate decreases, which is associated with a lowered capacity to resist perturbation and increased incidences of physical impairment, i.e., “frailty” [1]. Aging has also been associated with changes in gut microbiota (GM) composition and function in several studies [2,3,4,5,6]. Overall, the GM of frail older adults has a lower diversity and richness compared to that of the non-frail elderly [4,7], increased abundances of, e.g., *Enterobacteriaceae* [5,7], *Eggerthella lenta,* and *Eubacterium dolichum* [7], and a decreased abundance of *Faecalibacterium prausnitzii* [5,7]. However, perturbations in the GM of older adults are more likely to be associated with the health status, use of medication, and diet and other lifestyle factors rather than chronological aging per se [8]. In support of this, we have observed that in healthy older adults, specific GM and metabolomic signatures were associated with lower physical fitness [9]. Interestingly, Ghosh et al. [10] showed that adherence to a Mediterranean diet (high in plant-based food and relatively low in red meat, dairy products, and saturated fat) among non-frail and pre-frail subjects across five European countries was associated with improved frailty markers and accompanying changes in a range of microbial taxa.

Probiotics are defined as “live microorganisms that, when administered in adequate amounts, confer a health benefit on the host” [11]. Some studies show that probiotics induce changes in the GM by increasing potentially beneficial lactobacilli and bifidobacteria and inhibiting potential pathogens in older adults [12,13]. However, other studies and a meta-analysis conclude that, in general, probiotics do not appear to influence the GM composition to any larger extent in healthy adults [14,15]. Interestingly, even though Eloe-Fadrosh et al. [15] found that *Lacticaseibacillus rhamnosus* GG supplementation in older adults did not influence the overall GM composition, pronounced and consistent alterations in GM functional dynamics (gene expression) were induced by the probiotic [15].

Most studies investigating the effects of probiotics on the GM in older adults are relatively short-term (30 days–8 weeks), investigate the intake of single-strain probiotics, and/or are performed in relatively small cohorts [15,16,17,18,19]. However, despite these limitations, there are indications that supplementation of probiotics in the elderly might confer benefits, as exemplified by a meta-analysis showing that supplementation of *Bifidobacterium animalis* subsp. *lactis* HN019 in older adults increased polymorphonuclear (PMN) cell phagocytic capacity [20], and a study showing that, in a cohort of older adults with constipation, probiotic supplementation tended to decrease symptoms of constipation [21]. In younger adults receiving antibiotic treatment, the multi-strain probiotic HOWARU^®^ Restore (consisting of equal amounts of *Bifidobacterium animalis* subsp. *lactis* Bi-07, *B. animalis* subsp. *lactis* Bl-04, *Lactobacillus acidophilus* NCFM, and *Lacticaseibacillus paracasei* Lpc-37) has been found to reduce the risk of antibiotic-associated diarrhoea (AAD), *Clostridioides difficile*-associated diarrhoea (CDAD), and gastro-intestinal symptoms [22,23]. Likewise, a probiotic yogurt drink containing *Lacticaseibacillus casei* DN-114 001, *Lactobacillus delbrueckii* subsp. *bulgaricus,* and *Streptococcus thermophilus*, administered to hospitalised adults aged 50 years or more and taking antibiotics, has been found to significantly reduce the incidence of AAD as well as CDAD [24]. However, our knowledge of the effect of multi-strain probiotics on the GM composition, diversity, and function in healthy elderly is still sparse.

Short-chain fatty acids (SCFAs) such as acetate, propionate, and butyrate are produced in the colon from the breakdown of undigested dietary components like fibres and prebiotics, and play an important role in colonic health [25]. Faecal SCFA levels are generally reduced in the elderly compared to younger controls, but with pronounced intra-individual differences in both groups [26]. The frail elderly (hospitalised or nursing home residents) not only have pronounced lowered faecal SCFA levels compared to non-frail subjects of the same age group but also differ with respect to other metabolites such as, e.g., valerate and lipids [4,27]. Faecal metabolites from healthy older adults have been shown to decrease barrier function in vitro [28]. However, probiotic supplementation has been found to increase faecal SCFA levels in both experimental animals and human volunteers. Meanwhile, the effects of probiotic consumption on the faecal metabolic profile (including SCFA production) remain sparsely studied in older adults [29,30].

*C. difficile* infection remains a serious challenge in patients undergoing antibiotic treatment, especially in older adults, who are more susceptible to the disease as well showing higher morbidity and mortality resulting from the disease [31,32]. Age (65+ years of age) has been described as a risk factor for acquiring a *C. difficile* infection [32]. Nonetheless, it is not likely to be older age per se but instead other underlying conditions (immunosenescence, frailty) that increase the risk of acquiring *C. difficile* infection [33]. Relatively little is known about the *C. difficile* carriage rate of healthy, asymptotic older adults, with carriage rates from 1% up to ≈10% being reported [34,35].

In this randomised, double-blinded, placebo-controlled study, we investigated the effects of administrating a four-strain probiotic combination for 24 weeks on the levels of *C. difficile* along with the GM composition and faecal metabolome in 98 non-frail Danish older adults using high-throughput sequencing along with targeted (GC) and untargeted (^1^H NMR) profiling of faecal metabolites.

## 2. Materials and Methods

### 2.1. Study Design

With the overall objective of investigating whether multi-strain probiotic supplementation influences *C. difficile* faecal abundance, GM diversity, and faecal metabolites in the elderly, a 24-week randomised, double-blinded, placebo-controlled intervention where volunteers consumed either a four-strain probiotic combination or a placebo was carried out in the Copenhagen area of Denmark. Male and female volunteers aged 75 years or older were recruited for the study. Exclusion criteria were chronic bowel disease (e.g., Crohn’s disease or ulcerative colitis), severe immunosuppression, dementia, and terminal illness. Recruitment happened over a period of one year. A randomised block design was used to ensure an even distribution across the two intervention groups, excluding artefacts from different inclusion and sample storage times. Informed consent was collected from the participants before any intervention-related activities. This study was approved by the Danish Ethics Committee (protocol no. H-1-2014-051), carried out according to the principles set forth in the Declaration of Helsinki, and registered at clinicaltrials.gov as NCT02207140. A power calculation showed that by recruiting 140 subjects, with an expected drop-out rate of 10%, it would be possible to detect a difference in the numbers of *C. difficile* of log 0.6 ± 1.2 cells/g faeces, with a significance level of 0.05 and a power of 84%.

Before the onset of the intervention, the capsules containing either the probiotic or placebo were labelled with a random 5-digit number. The probiotic and placebo capsules used in this study were produced by DuPont (now IFF, Madison, WI, USA) and the randomisation list was generated by a person not involved in the study using the web-based random number generator http://www.randomization.com (accessed on 31 January 2014). Randomisation to the probiotic or placebo treatment was performed in a 1:1:1:1 ratio in blocks of 4 in a random order. Volunteers randomised to the probiotic arm consumed a capsule containing a total of 10^10^ colony forming units (CFU) of HOWARU^®^ Restore daily. The probiotic consists of equal amounts of four strains: *B. animalis* subsp. *lactis* Bi-07 (ATCC SD5220), *B. animalis* subsp. *lactis* Bl-04 (ATCC SD5219), *L. acidophilus* NCFM (ATCC 700396), and *L. paracasei* Lpc-37 (ATCC SD5275). Microcrystalline cellulose was used as an excipient, which was contained in the capsules consumed by volunteers randomised to the placebo arm. The capsules were identical in appearance and taste and all products were kept refrigerated (4 °C).

### 2.2. Compliance

Compliance with the intervention was determined by counting the returned capsules after the intervention. Furthermore, compliance was assessed by determining the presence of *B. animalis* subsp. *lactis* in faecal samples before and after the intervention using qPCR and species-specific primers, as described below.

### 2.3. Questionnaires

To investigate the influence of the probiotic intervention on digestive discomfort and bowel movements, questionnaire-based information was collected before, during (weeks 8 and 16), and after the intervention. Volunteers were asked to score their defecation frequency from 1 (<every second day) to 5 (>3 defecations daily), stool consistency from 1 (very hard) to 5 (very soft), and gastro-intestinal discomforts from 1 (no discomfort) to 5 (strong discomfort) [36].

### 2.4. Sample Collection and Pretreatment

Faecal samples were collected before and after the intervention. Volunteers were instructed to bring freshly collected samples, kept at 4 °C for no longer than 6 h, and they were then subsequently stored at −60 °C until analysis. The collected faecal samples were homogenised in sterile ultrapure water (Milli-Q^®^, Merck KGaA, Darmstadt, Germany) (1:2 *w*/*w*) and stored in aliquots (1.5 mL) at −60 °C until the extraction of DNA and metabolites.

### 2.5. Gut Microbiota Characterisation

DNA extraction was carried out using a PowerSoil extraction kit (Qiagen, Hilden, Germany), following the instructions of the manufacturer, but with minor modifications to ensure proper lysis of cells difficult to lyse. Prior to DNA extraction, the samples were placed into PowerBead tubes and heat treated at 65 °C for 10 min and then at 95 °C for 10 min. Subsequently, solution C1 was added and bead-beating performed in a FastPrep (MP Biomedicals, Santa Ana, CA, USA) using 3 cycles of 15 s each, at a speed of 6.5 m/s. The remaining DNA extraction procedure followed the manufacturer’s instructions.

Absolute quantification of *C. difficile* was performed by qPCR, as described previously [13]. Similarly, absolute quantification of *B. animalis* subsp. *lactis* was carried out by qPCR using species-specific primers, as described previously [37].

The GM composition (prokaryotic component) was determined by tag-encoded NextSeq-based (Illumina) high-throughput amplicon sequencing of the V3 region of the 16S rRNA gene, as previously described [9]. The raw dataset containing paired-end reads with corresponding quality scores was merged and trimmed, the dataset was purged of chimeric reads, and de novo zero-radius operational taxonomic units (zOTUs) were constructed, as previously described [9].

Community analysis was performed using phyloseq for R version 1.9.1 [38]. Alpha diversity metrics (observed species, Chao1, and Shannon index) were assessed while the UniFrac and Bray–Curtis dissimilarity indexes were used for assessing compositional differences (beta diversity) between samples. In total, the 188 samples had more than 46757 reads, and rarefaction was performed to this level, sustaining 26.4% of the total number of reads. Core species were defined as zOTUs present in more than 95% of the participants at the baseline. Bacterial taxa denoted as “unclassified” had no official taxonomy in the database, while clusters with more than one taxon are denoted “other”. Square brackets indicate taxa with a proposed taxonomy.

### 2.6. Faecal Metabolite Analysis

Concentrations of faecal SCFAs (acetic acid, propionic acid, butyric acid, and valeric acid) and branched-chain fatty acids (BCFAs; isobutyric acid, 2-methylbutyric acid, and isovaleric acid) were determined, as described previously [39]. Briefly, an internal standard (1 mL of 20 mM pivalic acid) and 5 mL of water were added to 1 g of the sample. After thorough mixing, the sample was centrifuged at 5000× *g* for 5 min. Following centrifugation, 250 μL of saturated oxalic acid solution was added to 500 μL of the supernatant, and the mixture was incubated at 4 °C for 60 min and then centrifuged at 16,000× *g* for 5 min, before 1 μL of the supernatant was used for analysis by GC-FID.

Faecal water for NMR-based metabolite analysis was extracted through the addition of 500 µL of 3xPBS (5.7 mM Na_2_HPO_4_, 24.3 mM NaH_2_PO_4_, 450 mM NaCl, pH 7.4) to 1.5 mL of homogenised faeces followed by ultracentrifugation (64,000× *g*, 2 h, 4 °C). A volume of 500 µL of supernatant was mixed with 100 µL of D_2_O containing 0.05% 3-(trimethylsilyl) propionic acid-d4 sodium salt (TSP) as an internal standard. The NMR measurements were performed at 298 K on a 600 MHz Avance III spectrometer (Bruker Biospins, Rheinstetten, Germany) operating at a ^1^H frequency of 600.13 MHz and equipped with a 5 mm broadband (BBO) probe. ^1^H NMR spectra were acquired using a standard 1D Noesy experiment with pre-saturation (Bruker “noesygppr1d” sequence), where the acquisition parameters were 32 scans, 64 K data points, a spectral width of 14.00 ppm, a recycle delay of 5 s, and an acquisition time of 3.90 s. All ^1^H spectra were processed with an exponential line-broadening of 0.8 Hz prior to Fourier transformation. Automatic metabolic deconvolution and quantifications of 46 metabolites were performed using the Bayesian AuTomated Metabolite ANalyser (BATMAN) R package, version 1.2.1.03 [40].

### 2.7. Statistical Analyses

Analyses of changes in defecation frequency, stool consistency, self-reported gastro-intestinal discomfort, concentrations of faecal metabolites including SCFA and BCFA, and alpha diversity, as well as univariate zOTU-wise models, were performed with the stats package within the statistical programming language R (v.3.5.1). The effects of the treatment were assessed by modelling the end-of-trial response as a function of the corresponding baseline value, probiotic treatment, and the interaction between the two. For individual zOTU models, log transformation with a pseudo count of 1 was used.

The differential zOTU abundance in relation to probiotic treatment was tested using a linear model with the treatment and individual as predictors, and we log transformed relative abundance as the response (including a pseudo count of 1 prior to normalisation). Likewise, the presence/absence was tested using logistic regression with the same predictors. For the microbiome, metabolome, and self-reported gastro-intestinal symptoms, the individual variables were analysed by ANOVA, where the variance was considered partitioned into variations related to the individual, treatment, and the residuals summing to 1, and visualised on a simplex.

Integration of the GM and faecal metabolome was performed by correlation analysis and visualised as heatmaps in ggplot2 (version 3.1.0), with the add-ons ggtern (version 3.1.0) and ggtree (version 1.14.6) used for visualisation.

## 3. Results

### 3.1. Impact of Probiotics on Self-Reported Gastro-Intestinal Discomfort

**Figure 1:**
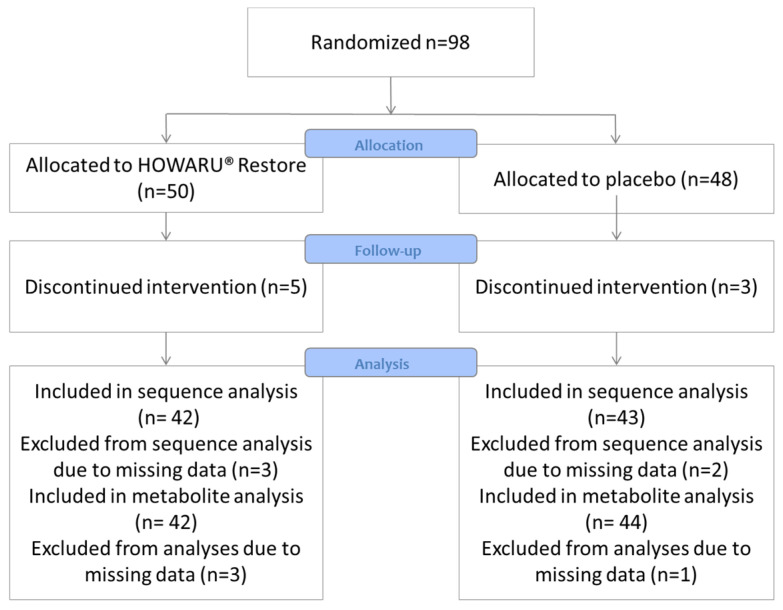
CONSORT diagram of the study.

A total of 98 volunteers were enrolled, of which 90 completed the study, with 45 in each group (Figure 1). The baseline characteristics of volunteers allocated to either the placebo or probiotic (HOWARU^®^ Restore) group were similar except for self-reported flatulence discomfort (Table 1). Counting of the returned capsules showed a 95% compliance for both the placebo and probiotic groups, while the absolute numbers of *B. animalis* subsp. *lactis* were increased for 96% of the volunteers in the probiotic group and 36% in the placebo group between the onset and completion of the intervention (Fisher’s exact test *p* < 0.001; Supplementary Figure S1A).

**Figure 2:**
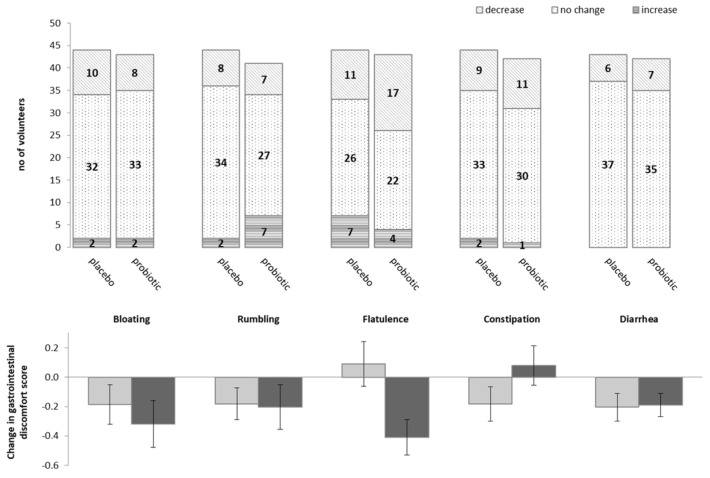
Distribution of volunteers reporting decrease, no change, and increase in discomfort of self-reported gastro-intestinal symptoms during intervention. Change in gastrointestinal discomfort score; light grey = placebo, dark grey = probiotic.

The probiotic mixture was well-tolerated and no differences in self-reported defecation frequency or stool consistency were found between the placebo and probiotic groups. As can be seen from Figure 2, 6–11 volunteers in the placebo and 7–17 volunteers in the probiotic group reported decreased levels of bloating, rumbling, flatulence, constipation, and diarrhoea during the 24 weeks of the intervention.

However, only for flatulence was there a larger reported decrease in discomfort after the probiotic intervention as compared to the placebo (*p* = 0.028; Figure 2). No other significant changes in discomfort scores compared to the baseline or between the treatment groups were observed.

### 3.2. C. difficile

Using species-specific qPCR, *C. difficile* was detected in the faecal samples from a few individuals (13 individuals the at baseline (5 from the probiotic group, 8 from the placebo group) and 20 individuals after the intervention (8 from the probiotic group and 12 from the placebo group)) but the levels were below the limit of quantification (2.01 log10 genomes/g) in all cases. The low levels of *C. difficile* both before and after intervention were supported by the fact that no zOTUs belonging to *C. difficile* were identified by 16S rRNA gene amplicon sequencing.

### 3.3. Faecal Microbiota of Danish Elderly

The total number of 16S rRNA gene sequence reads was 31,389,302, with an average of 166,964 ± 81,760 per sample, ranging from 46,757 to 618,436 and comprising a total of 13,614 zOTUs belonging to 682 species. At the baseline, the GM of the volunteers was dominated by the family *Ruminococcaceae,* covering 2306 zOTUs with a mean relative abundance of 33.7%, and *Lachnospiraceae* (946 zOTUs with a mean relative abundance of 32.4%) (Supplementary Table S1).

A total of 151 “core species”, defined as species identified in 95% of the baseline samples, were identified. They covered 41.8% of all reads and belonged primarily to two families: *Lachnospiraceae* and *Ruminococcaceae*. *Lachnospiraceae* members (115 zOTUs, with 92 unclassified beyond family) covered 27.2% of the total reads and, with the zOTUs identified to genus level, were assigned to *Blautia*, *Dorea*, and *Falcitimonas*. *Ruminococcaceae* (32 zOTUs, belonging to seven genera, and three unclassified) covered 13.4% of the total reads, with *Faecalibacterium* being particularly abundant (present in 96 samples out of 98), covering 2.2% of all reads. Additionally, three *Peptostreptococcaceae* zOTUs and one zOTU unclassified beyond the order *Clostridiales* were classified as core zOTUs (Table 2).

### 3.4. Effects of Intervention on Microbial Diversity and Composition

**Figure 3:**
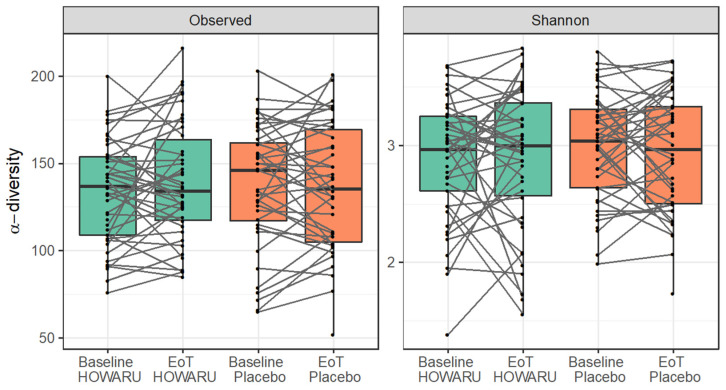
Number of observed species and Shannon diversity index before and after 24 weeks of intervention with HOWARU^®^ Restore or placebo. Lines indicate the individual development in alpha-diversity from baseline to end of trial (EoT).

**Figure 4:**
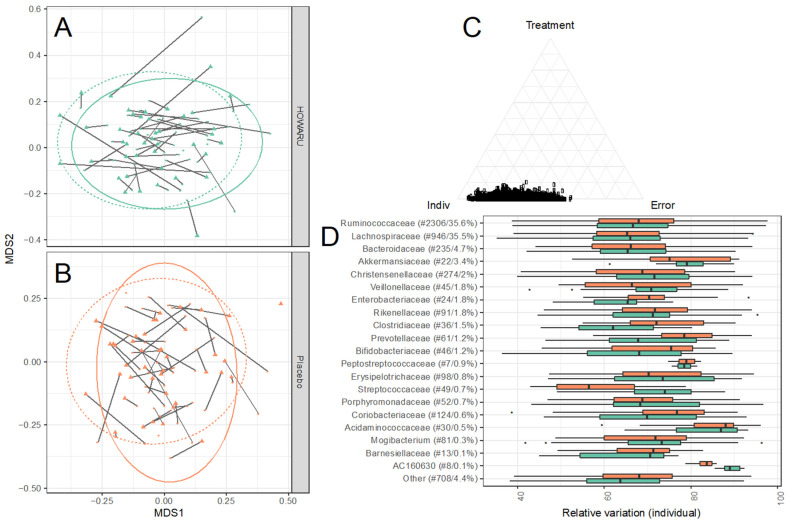
Intervention over 24 weeks with HOWARU^®^ Restore had no influence on overall gut microbiota composition structure (determined by 16S rRNA gene amplicon sequencing). (**A**,**B**) Bray–Curtis distance metric (NMDS), lines connects samples from baseline (dot) to 24 weeks (triangle) for the same individual; (**C**) simplex illustrating variance explained by individual, treatment, and residual for each OTU; (**D**) stability of the 10 most abundant families in the individuals undergoing intervention with HOWARU Restore (green) and placebo (orange), determined as relative sum of squares (SSQ).

Alpha diversity measures, as determined by the number of observed species and Shannon diversity index, were similar between treatments before and after the intervention (*p* = 0.2 and *p* = 0.8 for observed species and Shannon diversity index, respectively). showing that the intake of a multi-strain probiotic mixture over 24 weeks did not influence the alpha diversity. A high degree of intra-individual stability was observed (*p* < 10^−5^ for both alpha diversity measures) (Figure 3). In line with the pronounced intra-individual stability observed for alpha diversity measures, the overall GM composition (as determined by Bray–Curtis distance metrics) was largely determined by the individual, with 75% of the total variation in beta diversity accounted for by this factor (75% for the placebo arm and 74% for the HOWARU arm, Figure 4A–D). Similarly, in the weighted UniFrac distance analysis, the individual accounted for 72% of the variation. Smaller systematic changes were observed between the baseline and the end of the trial. However, the time point only accounted for 1.0% (*p* = 0.04) or 0.7% (*p* = 0.2) of the variation for the placebo and probiotic treatments, respectively, as determined by ADONIS analysis. A joint analysis revealed no differences in shift due to treatment (*p* = 0.23).

A variance analysis for each zOTU splitting the variance into treatment, intra-individual, and inter-individual variance showed a generally low treatment effect, but consistently high intra-individual variation (see Figure 4D) across all zOTUs. Interrogating the intra-individual results for the dominating 19 families revealed that approximately 70% of the total variance was due to the individual. The family *Acidaminococcaceae* (30 zOTUs accounting for 0.5% of the reads) was the most stable family, with an average of 84.5% (+/−8.5%) of the overall variation related to the individual (see Supplementary Table S2).

Overall, the GM of the enrolled elderly subjects showed remarkably high stability over the 24 weeks of the intervention, independent of the treatment, in terms of alpha diversity, overall composition, and the individual zOTU level.

Notably, the abundance of one zOTU (zOTU_1987) identified as belonging to either *B. animalis* subsp. *lactis* or *B. animalis* subsp. *animalis* (manual search using Ezbiocloud) increased during the intervention in the probiotic group from being present in 11% of the individuals at the baseline to 58% at the end of the intervention (q = 0.16). For the placebo group, this increase was from 7% at the baseline to 12% of the individuals at the end of the intervention. The HOWARU^®^ Restore probiotic mixture contained *B. animalis* subsp. *lactis* and the increase in the relative abundance of this zOTU in the probiotic group could likely be assigned to the intervention. Similarly, a zOTU assigned to *L. acidophilus* (also part of HOWARU^®^ Restore) was found to be significantly more abundant (q = 0.008) in the probiotic group following the intervention (Supplementary Figure S1B), again underlining that the administered probiotics could be detected in the GM of the older adults receiving the probiotic mixture.

### 3.5. Effects of Probiotics on Metabolite Composition

**Figure 5:**
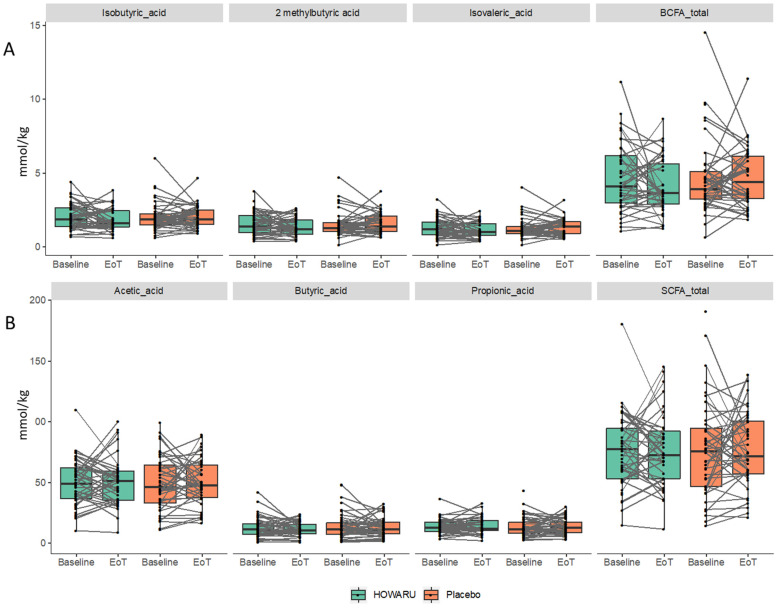
Faecal concentrations of branched-chain fatty acids (**A**) and short-chain fatty acids (**B**) after 24 weeks of intervention with HOWARU^®^ Restore or placebo. Lines indicate the individual development in α-diversity from baseline to end of trial (EoT).

Following baseline correction, no differences in total or individual SCFAs, i.e., acetic, propionic, or butyric acid, or in BCFAs (isobutyric, isovaleric, or 2-methylbutyric acid) were observed between treatment groups at the baseline or after the placebo or probiotic intervention (Figure 4, Supplementary Tables S3 and S4). A total of 46 known faecal metabolites were identified from the 1H-NMR spectra obtained (see Supplementary Table S4). Most of the identified metabolites are amino acids or products of amino acid breakdown (21 metabolites), organic acids (8 metabolites), and metabolites with a plant or dietary origin (4 metabolites). With the probiotic intervention, six metabolites obtained nominal significance after the intervention, namely glycine, lysine, fumarate, glycerophosphorylcholine, ribose, and arabinose. However, the effect size was small, and none passed false discovery rate correction (q > 0.1). Overall, the faecal metabolome was also observed for the GM to be remarkably stable during the 24 weeks of the intervention (Figure 5 and Supplementary Tables S3 and S4).

**Figure 6:**
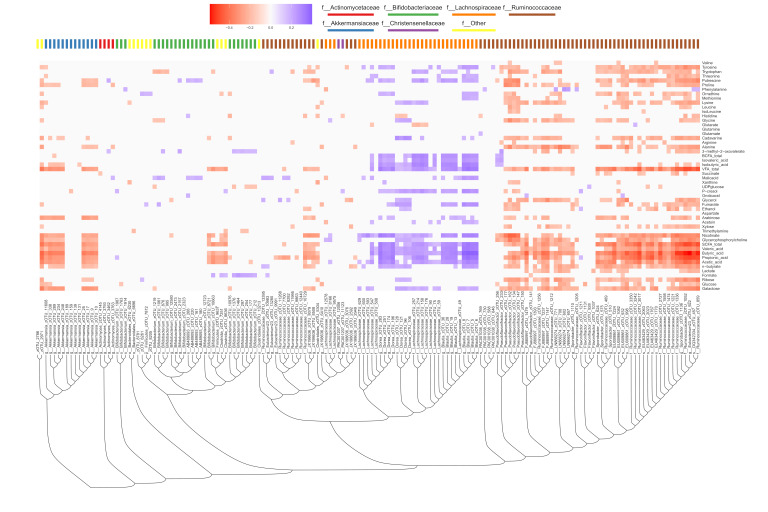
Heatmap of zOTUs and metabolites.

To investigate links between the GM and metabolites, all zOTUs and metabolites were correlated. A total of 159 zOTUs and 53 metabolites (from GC-FID and NMR) had a correlation coefficient numerically larger than r = 0.16 and are visualised in Figure 6. Interestingly, most correlations are negative, but a relatively large proportion of zOTUs belonging to the Lachnospiraceae family is positively correlated with mainly the SCFA and BCFA concentrations but also a range of amino acids (Figure 6).

## 4. Discussion

Overall, the GM of the enrolled elderly subjects showed remarkably high stability over the 24 weeks of the intervention, independent of the treatment, in terms of the alpha diversity, overall composition, and individual zOTU level.

Aging has been associated with changes in the GM composition and function in several studies [2,4,5,6]. However, these changes in the GM structure of older adults are probably associated with the health status, use of medication, and diet and other lifestyle factors rather than chronological aging per se [8]. A loss of microbial diversity has been associated with frailty [4,7], and while a causal relationship remains to be established between decreased microbial diversity and increased frailty in the elderly, a better understanding of the dynamics of the aging GM and ways to influence its composition, e.g., by probiotics, may potentially be part of the key for improving health [10,41]. This study is the first to investigate how the long-term (24 weeks) intake of a four-strain probiotic, HOWARU Restore, affects the GM and faecal metabolome of healthy older adults in a double-blinded, randomised, placebo-controlled study.

Several studies have shown that probiotics are able to lower flatulence in patients suffering from irritable bowel disease as well as improve bowel function [42,43,44]. Furthermore, in older adults, probiotic consumption improved symptoms of functional constipation [21,45] and, in frail elderly, probiotics increased “ideal stools”, thereby improving bowel habits [46]. We found that the four-strain probiotic mixture used in this study was able to decrease self-reported flatulence discomfort in the elderly, while no changes in defecation frequency or stool consistency were observed. Previously a combination of *Lb. acidophilus* NCFM and *B. animalis* subsp. *lactis* Bi-07, two of the strains used in this study, was shown to decrease abdominal bloating in patients with functional bowel disorders [47], which, in combination with our findings, suggests that the four-strain probiotic used in the current study may help ease digestive flatulence, although this proposal may related to the increased baseline levels of flatulence in the probiotic group. Nevertheless, it may be worthwhile to explore this in a larger cohort targeting (elderly) individuals suffering from functional bloating.

The GM of the elderly in the current study was dominated by *Firmicutes* members (Table 2 and Supplementary Table S1) and differed from the Irish ELDERMET cohort, where the microbiome was dominated by *Bacteroidetes* [3,4], though with very large inter-individual differences. The majority of core species in the present study belonged to *Ruminococcaceae* and *Lachnospiraceae,* in total constituting 66.1% of the mean relative abundance of species shared across 95% of the individuals (Table 2 and Supplementary Table S1). As such, it showed high similarity with the microbiome of the Italian elderly, which a previous study found to be dominated by the same taxa [48], as well as a cohort of older Danish adults with an age on average 10 years lower than in the present study [9]. Possibly, this can be ascribed to different dietary patterns between the three countries. As can be seen from Figure 2 and Figure 3, the impacts of the consumption of the four-strain probiotic mixture on GM diversity and composition were minor, which agreed with the findings of studies using single-strain probiotics [15,18,49].

Age is considered a risk factor with regard to developing CDAD following antibiotic treatment. Consumption of probiotics has been reported to be effective in reducing the risk of *C. difficile* infection [50]. The four-strain probiotic used in the current study was previously shown to lower the risk of CDAD in a hospital setting [22,23]; furthermore, *L. acidophilus* NCFM combined with *L. rhamnosus* HN001 decreased the numbers of *C. difficile* in asymptomatic carriers in a nursing home setting [13]. The rate of asymptomatic carriers in the community-dwelling elderly has been reported to be between 1 and 10% [13,34,35,51,52]. However, in the present study, *C. difficile* was detected in 13 and 22% of the subjects at the baseline and by the end of the study, respectively. Importantly, all samples were below the limit of quantification by qPCR (2.01 log_10_ genomes/g), indicating a low abundance of *C. difficile* in healthy Danish older adults. Although the power calculation indicated that 140 subjects would be needed to observe a possible difference, this number was not met due to time constraints and recruitment challenges. Nonetheless, considering the low incidence and low abundance of *C. difficile*, it is unlikely that it would have changed the outcome even if the number had been reached.

While the importance of SCFAs, mainly produced by fermentation of carbohydrates, in colonic health is well-known, little has been investigated regarding the role of BCFAs in the gut. BCFAs are commonly used as biomarkers of colonic protein fermentation leading to the concomitant production of other potentially toxic protein fermentation products such as ammonia, phenol, and H_2_S, which can cause cell damage in the intestine [53,54]. Nagpal et al. [30] found that probiotic consumption increased faecal SCFA levels, but other studies found no effect [17]. In the present study, four-strain probiotic consumption for 24 weeks had no effect on faecal concentrations in terms of either the SCFA or BCFA level (Figure 5, Supplementary Tables S3 and S4). The stability of faecal metabolites correlates with the stability of the faecal microbiota composition. Although positive correlations with mainly the SCFA and BCFA concentrations were observed for zOTUs belonging to the *Lachnospiraceae* family, one of the main core families in this study cohort, *Lachnospiraceae*’s ability to produce SCFA and BCFA was in agreement with earlier reports [55].

In mice, an age-related response of the faecal metabolome to probiotic administration has been reported [56], and probiotic administration has been found to influence both the GM composition and the faecal metabolome [57]. However, only scarce information is available regarding the effects of probiotic consumption on the faecal metabolic profile of older adults. When using ^1^H NMR spectroscopy profiling and targeted GC-FID, the current study did not find probiotic-induced changes in the overall faecal metabolome profile of the elderly, nor were the identified compounds affected. In line with the findings for the GM, we observed high inter-individual stability of the faecal metabolome throughout the 24 weeks of probiotic administration.

## 5. Conclusions

In conclusion, the GM as well as the faecal metabolome of healthy Danish older adults showed remarkably stability over 24 weeks, even when exposed to a daily dose of the multi-strain probiotic mixture HOWARU Restore. This correlated with the observation that most volunteers in both study groups did not experience a change in self-reported digestive symptoms. Only flatulence was positively influenced by probiotic supplementation as compared to the placebo.

## Figures and Tables

**Table 1:** Baseline characteristics of volunteers who completed the trial with regards to age, sex, medication, defecation frequency, stool consistency, and GI symptoms. Age is shown as average (age range shown in brackets), while sex, medication, defecation frequency, stool consistency, and GI symptoms are shown as number of volunteers (with the percentage in brackets). *p*-values, as determined by 2-sided *t*-test, between placebo and probiotic groups are shown.

		Placebo *n* = 45	Probiotics *n* = 45	*p*-Value
Age (years)	Median [IQR]	77 (76–80)	79 (76–82)	0.15
Sex	Male	18 (40%)	15 (33%)	0.66
	Female	27 (60%)	30 (67%)	
Medication ^#^	Yes	28 (62%)	28 (62%)	1.00
	No	17 (38%)	17 (38%)	
Defecation frequency	Less than every second day	2 (5%)	0 (0%)	0.42
	Every second day	4 (10%)	5 (12%)	
	Once daily	19 (45%)	26 (61%)	
	1–2 times daily	7 (17%)	5 (12%)	
	2–3 times daily	9 (21%)	5 (12%)	
	More than 3 times daily	1 (2%)	2 (5%)	
Stool consistency	Very hard	4 (9%)	2 (4%)	0.27
	Hard	7 (16%)	11 (24%)	
	Neither hard nor soft	23 (52%)	18 (40%)	
	Soft	10 (23%)	11 (24%)	
	Very soft	0 (0%)	3 (7%)	
Bloating	No discomfort	34 (77%)	35 (78%)	0.87
	Mild to moderate discomfort	8 (18%)	7 (16%)	
	Strong to very strong discomfort	2 (5%)	3 (7%)	
Rumbling	No discomfort	34 (77%)	35 (81%)	0.82
	Mild to moderate discomfort	8 (18%)	7 (16%)	
	Strong to very strong discomfort	2 (5%)	1 (2%)	
Flatulence	No discomfort	31 (69%)	18 (41%)	0.03
	Mild to moderate discomfort	9 (20%)	16 (36%)	
	Strong to very strong discomfort	5 (11%)	10 (23%)	
Constipation	No discomfort	35 (80%)	33 (75%)	0.36
	Mild to moderate discomfort	6 (14%)	10 (23%)	
	Strong to very strong discomfort	3 (7%)	1 (2%)	
Diarrhoea	No discomfort	36 (84%)	37 (84%)	0.246
	Mild to moderate discomfort	4 (9%)	1 (2%)	
	Strong to very strong discomfort	3 (7%)	6 (14%)	
Intestinal pain, after meal	No discomfort	41 (91%)	43 (96%)	0.68
	Mild to moderate discomfort	4 (9%)	2 (4%)	
	Strong to very strong discomfort	0 (0%)	0 (0%)	
Intestinal pain, general	No discomfort	45 (100%)	44 (100%)	1.00
	Mild to moderate discomfort	0 (0%)	0 (0%)	
	Strong to very strong discomfort	0 (0%)	0 (0%)	

# Medication defined as all types of orally consumed prescription drugs.

**Table 2:** Average relative abundances along with minimum and maximum observations of core species shared across 95% of the baseline samples in the faecal microbiome of older Danish adults (at baseline), as determined by 16S rRNA gene amplicon sequencing (*n* = 98). No. of zOTUs—number of zOTUs representing the given taxa. No. of samples—number of samples where the given taxon was observed. No. of samples (%)—percentage of samples where the given taxon was observed.

Phylum	Class	Order	Family	Genus	Species	No. of zOTUs	No. of Samples	No. of Samples (%)	Maximum Reads (%)	Mean Reads (%)
*Actinobacteria*	*Coriobacteriia*	*Coriobacteriales*	*Coriobacteriaceae*			35	95	97	0.4	0.0
*Bacteroidetes*	*Bacteroidia*	*Bacteroidales*	*Bacteroidaceae*	*Bacteroides*	*Bacteroides*	78	98	100	19.7	2.2
*Bacteroidetes*	*Bacteroidia*	*Bacteroidales*	*Rikenellaceae*	*Alistipes*	*Alistipes*	52	98	100	6.6	0.9
*Bacteroidetes*	*Bacteroidia*	*Bacteroidales*	*Bacteroidaceae*	*Bacteroides*		148	95	97	15.2	1.2
*Firmicutes*	*Clostridia*	*Clostridiales*	*Lachnospiraceae*			725	98	100	45.3	23.0
*Firmicutes*	*Clostridia*	*Clostridiales*	*Ruminococcaceae*	*Faecalibacterium*		115	98	100	35.8	7.8
*Firmicutes*	*Clostridia*	*Clostridiales*	*Lachnospiraceae*	*Blautia*		73	98	100	53.6	6.8
*Firmicutes*	*Clostridia*	*Clostridiales*	*Ruminococcaceae*			928	98	100	12.8	4.5
*Firmicutes*	*Clostridia*	*Clostridiales*				367	98	100	6.7	2.8
*Firmicutes*	*Clostridia*	*Clostridiales*	*Ruminococcaceae*	*Sporobacter*		148	98	100	8.3	1.9
*Firmicutes*	*Clostridia*	*Clostridiales*	*Ruminococcaceae*	*Oscillibacter*		63	98	100	15.9	1.8
*Firmicutes*	*Clostridia*	*Clostridiales*	*Lachnospiraceae*	*Dorea*	*Dorea*	13	98	100	3.9	0.9
*Firmicutes*						97	98	100	3.5	0.5
*Firmicutes*	*Clostridia*	*Clostridiales*	*Lachnospiraceae*	*Lachnospira*		12	98	100	8.4	0.4
*Firmicutes*	*Clostridia*	*Clostridiales*	*Lachnospiraceae*	*Falcatimonas*	*Falcatimonas*	4	98	100	2.3	0.4
*Firmicutes*	*Clostridia*	*Clostridiales*	*Ruminococcaceae*	*Pseudoflavonifractor*		60	98	100	1.6	0.3
*Firmicutes*	*Clostridia*	*Clostridiales*	*Ruminococcaceae*	*Agathobaculum*		25	98	100	3.6	0.3
*Firmicutes*	*Clostridia*	*Clostridiales*	*Ruminococcaceae*	*Ruthenibacterium*	*Ruthenibacterium*	4	98	100	1.2	0.1
*Firmicutes*	*Clostridia*	*Clostridiales*	*Mogibacterium*	*PAC001168*		14	98	100	0.6	0.1
*Firmicutes*	*Clostridia*	*Clostridiales*	*Ruminococcaceae*	*Subdoligranulum*	DQ800172	4	97	99	13.1	1.7
*Firmicutes*	*Clostridia*	*Clostridiales*	*Ruminococcaceae*	*Faecalibacterium*	GL538271	4	97	99	10.8	1.4
*Firmicutes*	*Clostridia*	*Clostridiales*	*Ruminococcaceae*	*Subdoligranulum*		11	97	99	5.7	1.1
*Firmicutes*	*Clostridia*	*Clostridiales*	*Peptostreptococcaceae*			4	97	99	5.4	0.7
*Firmicutes*	*Clostridia*	*Clostridiales*	*Ruminococcaceae*	*Pseudoflavonifractor*	LN866274	4	97	99	0.8	0.1
*Firmicutes*	*Clostridia*	*Clostridiales*	*Lachnospiraceae*	PAC000195		1	97	99	0.2	0.1
*Firmicutes*	*Clostridia*	*Clostridiales*	*Clostridiaceae*	*Clostridium*		23	96	98	14.2	1.3
*Firmicutes*	*Clostridia*	*Clostridiales*	*Ruminococcaceae*	*Oscillibacter*	PAC001129	2	96	98	6.9	0.8
*Firmicutes*	*Clostridia*	*Clostridiales*	*Ruminococcaceae*	PAC000672		9	96	98	1.8	0.3
*Firmicutes*	*Clostridia*	*Clostridiales*	*Christensenellaceae*	PAC001207		50	96	98	1.6	0.3
*Firmicutes*	*Clostridia*	*Clostridiales*	*Ruminococcaceae*	*Ruminococcus*		48	96	98	1.5	0.2
*Firmicutes*	*Clostridia*	*Clostridiales*	*Lachnospiraceae*	PAC000195	PAC000195	1	96	98	0.3	0.1
*Firmicutes*	*Clostridia*	*Clostridiales*	*Ruminococcaceae*	*Eubacterium23*		36	95	97	43.7	2.7
*Firmicutes*	*Clostridia*	*Clostridiales*	*Ruminococcaceae*	*Paludicola*		48	95	97	0.9	0.1
*Firmicutes*	*Clostridia*	*Clostridiales*	*Ruminococcaceae*	*Subdoligranulum*	DQ793991	20	95	97	0.5	0.1
*Firmicutes*	*Clostridia*	*Clostridiales*	*Christensenellaceae*	PAC001207	EU472329	4	94	96	2.7	0.3
*Firmicutes*	*Clostridia*	*Clostridiales*	*Christensenellaceae*			44	94	96	0.5	0.1
*Firmicutes*	*Clostridia*	*Clostridiales*	*Ruminococcaceae*	*Oscillibacter*	JPJG	6	93	95	5.5	0.8
*Firmicutes*	*Bacilli*	*Lactobacillales*	*Streptococcaceae*	*Streptococcus*		35	93	95	11.8	0.4
*Firmicutes*	*Clostridia*	*Clostridiales*	*Peptostreptococcaceae*	*Clostridioides*	*Clostridium*	3	93	95	1.6	0.1
*Firmicutes*	*Clostridia*	*Clostridiales*	*Lachnospiraceae*	*Shuttleworthia*		4	93	95	0.3	0.1
*Firmicutes*	*Clostridia*	*Clostridiales*	*Mogibacterium*			15	93	95	0.3	0.0
Unclassified						168	98	100	3.5	0.7

## Data Availability

The 16S rRNA gene amplicon sequence dataset is available from the BioProject database (https://www.ncbi.nlm.nih.gov/bioproject, accessed on 19 March 2024) with accession number PRJNA923577. An example of 1H NMR data acquired on the human faecal samples is shown in Supplementary Figure S2. The full 1H NMR dataset as well as associated metadata are available upon request by contacting the corresponding author.

## Supplementary Materials

The following supporting information can be downloaded at https://www.mdpi.com/article/10.3390/microorganisms12040796/s1.
