# Supplementary material for: Randomised, Placebo-Controlled Investigation of the Impact of Probiotic Consumption on Gut Microbiota Diversity and the Faecal Metabolome in Seniors
Source: Microorganisms. 2024 Apr 15;12(4):796. doi: 10.3390/microorganisms12040796 (PMC11052279; doi:10.3390/microorganisms12040796)

Supplementary Table S1. The most abundant bacterial families in the faecal microbiome of older Danish adults (at baseline), as determined by 16S rRNA gene amplicon sequencing (n = 98). No. of zOTUs; number of zOTUs representing the given taxa. No. of samples; number of samples where the given taxa was observed. No. of samples (%); percentage of samples, where the given taxa was observed.

Phylum	Class	Order	Family	No. of zOTUs	No. of samples (%)	Maximum reads (%)	Mean reads (%)
Firmicutes	*Clostridia*	*Clostridiales*	*Ruminococcaceae*	2306	100.00	67.95	33.71
Firmicutes	*Clostridia*	*Clostridiales*	*Lachnospiraceae*	946	100.00	94.07	32.37
Verrucomicrobia	*Verrucomicrobiae*	*Verrucomicrobiales*	*Akkermansiaceae*	22	90.82	36.53	3.86
Bacteroidetes	*Bacteroidia*	*Bacteroidales*	*Bacteroidaceae*	235	100.00	29.91	3.45
Firmicutes	*Clostridia*	*Clostridiales*		367	100.00	6.72	2.78
Firmicutes	*Negativicutes*	*Veillonellales*	*Veillonellaceae*	45	88.78	31.84	2.22
Proteobacteria	*Gammaproteobacteria*	*Enterobacterales*	*Enterobacteriaceae*	24	91.84	64.61	2.16
Firmicutes	*Clostridia*	*Clostridiales*	*Christensenellaceae*	274	100.00	7.71	1.86
Firmicutes	*Clostridia*	*Clostridiales*	*Clostridiaceae*	36	97.96	14.77	1.42
Actinobacteria	*Actinobacteria*	*Bifidobacteriales*	*Bifidobacteriaceae*	46	94.90	12.99	1.22
Bacteroidetes	*Bacteroidia*	*Bacteroidales*	*Rikenellaceae*	91	100.00	11.80	1.16
Bacteroidetes	*Bacteroidia*	*Bacteroidales*	*Prevotellaceae*	61	70.41	30.99	1.02
Firmicutes	*Clostridia*	*Clostridiales*	*Peptostreptococcaceae*	7	98.98	6.96	0.85
Firmicutes	*Erysipelotrichi*	*Erysipelotrichales*	*Erysipelotrichaceae*	98	100.00	4.97	0.78
Firmicutes	*Bacilli*	*Lactobacillales*	*Streptococcaceae*	49	100.00	12.16	0.70
Unclassified Bacteria				168	100.00	3.52	0.67
Actinobacteria	*Coriobacteriia*	*Coriobacteriales*	*Coriobacteriaceae*	124	100.00	2.94	0.58
Firmicutes				97	100.00	3.48	0.51
Firmicutes	*Negativicutes*	*Acidaminococcales*	*Acidaminococcaceae*	30	79.59	9.28	0.48
Bacteroidetes	*Bacteroidia*	*Bacteroidales*	*Porphyromonadaceae*	52	97.96	3.34	0.36
Firmicutes	*Clostridia*	*Clostridiales*	*Mogibacterium*	81	100.00	2.73	0.30
Bacteroidetes	*Bacteroidia*	*Bacteroidales*	*Barnesiellaceae*	13	81.63	2.82	0.12
Bacteroidetes	*Bacteroidia*	*Bacteroidales*	AC160630	8	18.37	3.34	0.10
Proteobacteria	*Deltaproteobacteria*	*Desulfovibrionales*	*Desulfovibrionaceae*	19	94.90	1.04	0.08
Proteobacteria	*Gammaproteobacteria*	*Enterobacterales*		8	32.65	4.35	0.08
Firmicutes	*Bacilli*	*Lactobacillales*	*Lactobacillaceae*	18	60.20	2.42	0.08
Bacteroidetes	*Bacteroidia*	*Bacteroidales*		41	90.82	0.41	0.07
Proteobacteria	*Betaproteobacteria*	*Burkholderiales*	*Sutterellaceae*	22	67.35	0.84	0.04
Bacteroidetes	*Bacteroidia*	*Bacteroidales*	*Odoribacteraceae*	23	79.59	0.33	0.03
Verrucomicrobia	*Verrucomicrobiae*	*Verrucomicrobiales*		1	58.16	0.25	0.03

Verrucomicrobia				4	44.90	0.35	0.02
Firmicutes	*Clostridia*	*Clostridiales*	*Peptococcaceae*	6	38.78	0.27	0.02
Proteobacteria				2	36.73	0.44	0.02
Proteobacteria	*Alphaproteobacteria*	*Rhodospirillales*	*Rhodospirillaceae*	13	46.94	0.41	0.01
Synergistetes	*Synergistia*	*Synergistales*	*Synergistaceae*	3	18.37	1.12	0.01
Actinobacteria				17	85.71	0.16	0.01
Firmicutes	*Clostridia*	*Clostridiales*	*Dehalobacterium*	9	66.33	0.04	0.01
Tenericutes	*Mollicutes*	*PAC001057*	*PAC001057*	9	47.96	0.19	0.01
Actinobacteria	*Actinobacteria*	*Actinomycetales*	*Actinomycetaceae*	11	79.59	0.05	0.01
Proteobacteria	*Gammaproteobacteria*	*Pasteurellales*	*Pasteurellaceae*	7	40.82	0.11	0.01
Bacteroidetes				1	50.00	0.05	0.00
Cyanobacteria	*Vampirovibrio*	FR888536	FR888536	5	27.55	0.11	0.00
Firmicutes	*Clostridia*	*Clostridiales*	*Thermincola*	6	33.67	0.05	0.00
Tenericutes	*Mollicutes*	PAC001057	PAC000197	2	19.39	0.07	0.00
*Saccharibacteria*_TM7	*Saccharimonas*	*Saccharimonas*	*Saccharimonas*	4	37.76	0.02	0.00
Firmicutes	*Negativicutes*	*Hydrogenispora*	*Hydrogenispora*	4	33.67	0.01	0.00
Proteobacteria	*Betaproteobacteria*	*Burkholderiales*	*Oxalobacteraceae*	2	16.33	0.01	0.00
Actinobacteria	*Actinobacteria*			1	13.27	0.01	0.00
Firmicutes	*Bacilli*	*Bacillales*	*Gemella*	1	14.29	0.01	0.00
Tenericutes	*Mollicutes*	*Acholeplasmatales*	*Acholeplasmataceae*	1	8.16	0.01	0.00
Proteobacteria	*Gammaproteobacteria*			1	13.27	0.01	0.00

Supplementary Table S2: Stability reflected as variance explained (R^2) from ANOVA modelling with *individual* as factor for each zOTU. Summary statistics on the level of the top 20 most abundant families. SD, standard deviation.

**Family (#zOTUs within / overall relative abundance)**	**Average R^2**	**Min R^2**	**Max R^2**	**SD R^2**
AC160630 (#8/0.1%)	86.3	78.7	92.4	3.9
Acidaminococcaceae (#30/0.5%)	84.6	59.5	96.1	8.5
Peptostreptococcaceae (#7/0.9%)	78.7	74.7	82.4	2.4
Akkermansiaceae (#22/3.4%)	77.9	52.5	91.2	9.2
Prevotellaceae (#61/1.2%)	74.5	46.4	94.1	10.9
Erysipelotrichaceae (#98/0.8%)	72.7	46.9	94.6	12.3
Coriobacteriaceae (#124/0.6%)	72.6	38.7	92.9	11.8
Mogibacterium (#81/0.3%)	71.0	41.9	96.6	11.2
Rikenellaceae (#91/1.8%)	70.2	44.5	95.5	11.4
Christensenellaceae (#274/2%)	69.5	39.8	94.2	12.2
Porphyromonadaceae (#52/0.7%)	69.3	43.1	96.8	12.6
Veillonellaceae (#45/1.8%)	69.1	42.9	92.0	11.9
Bifidobacteriaceae (#46/1.2%)	68.8	36.4	89.6	13.5
Clostridiaceae (#36/1.5%)	67.8	45.2	90.4	11.1
Barnesiellaceae (#13/0.1%)	67.2	44.9	82.8	10.5
Ruminococcaceae (#2306/35.6%)	66.9	38.6	97.7	11.4
Bacteroidaceae (#235/4.7%)	66.5	42.0	94.2	11.0
Enterobacteriaceae (#24/1.8%)	66.4	48.0	93.5	9.2
Streptococcaceae (#49/0.7%)	65.7	42.8	88.0	12.0
Lachnospiraceae (#946/35.5%)	65.3	35.1	94.4	10.5

Supplementary Table S3. Fecal short chain and branched chain fatty acids (SCFA and BCFA) concentrations at baseline and after treatment with placebo HOWARU© determined by targeted GC-FID.

	**Placebo, Baseline**			**Treatment**				**Probiotic Baseline**			**Treatment**			
(mmol/kg)	Mean	Min.	Max.	Mean	Min.	Max.	p-value	Mean	Min.	Max.	Mean	Min.	Max.	q-value[2]
Acetic acid	45.7	8.0	96.4	47.3	13.6	86.7	0.72	46.6	7.4	106.8	48.1	5.8	97.4	0.73
Propionic acid	12.7	1.7	42.4	13.5	2.4	29.3	0.63	13.2	3.0	35.9	13.7	1.3	32.3	0.74
Butyric acid	13.6	0.7	48.0	12.6	1.3	32.3	0.65	12.7	0.5	41.6	11.6	0.5	23.4	0.48
Valeric acid	2.0	0	8.3	12.6	0	4.8	0.69	1.7	0	3.4	1.6	0	3.6	0.64
SCFA[1]	71.9	10.4	186.8	73.4	17.5	134.7	0.85	72.6	10.9	176.7	73.4	7.6	141.7	0.90
Isobutyric acid	1.8	0.4	5.8	13.8	0.7	4.5	0.95	1.9	0.5	4.2	1.6	0.4	3.6	0.13
2-methylbutyric acid	1.4	0	4.6	1.5	0.5	3.6	0.65	1.4	0.2	3.7	1.2	0.3	2.5	0.11
Isovaleric acid	1.1	0	3.9	1.2	0.4	3.1	0.65	1.2	0	3.1	1.0	0.3	2.3	0.14
BCFA[2]	4.4	0.4	14.3	4.5	1.6	137.4	0.76	4.4	0.9	10.9	3.7	1.0	8.5	0.12

[1]Sum of major SCFAs, acetic acid, propionic acid and butyric acid. [2]Sum of BCFAs isobutyruc acid, 2-methylbutyric acid and isovaleric acid

[2]q-values (FDR-adjusted p-values)

Supplementary Table S4. Relative concentrations (arbitrary units) of faecal metabolites determined by NMR at baseline and after intervention. Metabolites are grouped by their functional classification.

Metabolic group	Compound	Placebo Baseline		Placebo Treatment		Probiotic Baseline		Probiotic Treatment		q-value[1]
		Mean	SE	Mean	SE	Mean	SE	Mean	SE	
Amine metabolism	Cadavarine	0.017	0.003	0.017	0.003	0.016	0.002	0.013	0.001	0.76
	Putrescine	0.015	0.002	0.016	0.003	0.014	0.002	0.015	0.003	0.90
	Trimethylamine	0.002	0.000	0.003	0.001	0.004	0.001	0.002	0.001	0.24
Amino acid metabolism	3-methyl-2-oxovalerate	0.024	0.003	0.034	0.004	0.027	0.004	0.027	0.003	0.24
	Alanine	0.258	0.033	0.308	0.047	0.332	0.047	0.280	0.042	0.24
	Arginine	0.029	0.006	0.029	0.006	0.041	0.010	0.025	0.004	0.40
	Aspartate	0.052	0.008	0.055	0.012	0.040	0.008	0.033	0.006	0.87
	Glutamate	0.089	0.009	0.115	0.016	0.115	0.015	0.090	0.011	0.24
	Glutamine	0.032	0.005	0.039	0.007	0.040	0.006	0.029	0.004	0.24
	Glutarate	0.021	0.003	0.029	0.005	0.026	0.004	0.021	0.002	0.24
	Glycine	0.117	0.018	0.173	0.027	0.204	0.025	0.146	0.027	0.12
	Histidine	0.003	0.000	0.004	0.001	0.003	0.001	0.003	0.001	0.68
	Iso Leucine	0.118	0.012	0.149	0.022	0.159	0.021	0.128	0.017	0.24
	Leucine	0.104	0.011	0.137	0.018	0.143	0.017	0.130	0.017	0.58
	Lysine	0.047	0.009	0.053	0.010	0.041	0.007	0.034	0.005	0.54
	Methionine	0.059	0.011	0.076	0.013	0.066	0.008	0.065	0.012	0.48
	Ornethine	0.113	0.016	0.131	0.015	0.136	0.025	0.119	0.019	0.48
	Phenylalanine	0.071	0.012	0.083	0.015	0.080	0.010	0.070	0.011	0.54
	Proline	0.055	0.013	0.081	0.016	0.092	0.023	0.054	0.011	0.24
	Threonine	0.063	0.010	0.087	0.012	0.088	0.014	0.085	0.014	0.51
	Tryptophan	0.008	0.001	0.008	0.002	0.010	0.002	0.008	0.001	0.53
	Tyrosine	0.082	0.011	0.092	0.013	0.110	0.017	0.098	0.014	0.42
	Valine	0.110	0.012	0.129	0.021	0.144	0.020	0.111	0.016	0.28
Carbohydrate metabolism	Acetoin	0.076	0.021	0.125	0.035	0.058	0.018	0.052	0.020	0.44
	Arabinose	0.038	0.007	0.066	0.010	0.072	0.019	0.051	0.016	0.24

	Ethanol	0.094	0.019	0.089	0.019	0.082	0.014	0.083	0.018	0.92
	Formate	0.014	0.002	0.026	0.012	0.026	0.009	0.010	0.002	0.24
	Galactose	0.039	0.009	0.052	0.012	0.028	0.006	0.030	0.006	0.54
	Glucose	0.017	0.003	0.029	0.007	0.018	0.004	0.014	0.003	0.24
	Glycerol	0.268	0.047	0.355	0.062	0.488	0.129	0.282	0.044	0.24
	Lactate	0.138	0.034	0.114	0.014	0.152	0.026	0.149	0.037	0.90
	Orotic acid	0.006	0.001	0.005	0.001	0.006	0.001	0.005	0.001	0.76
	Ribose	0.333	0.079	0.294	0.042	0.288	0.042	0.214	0.032	0.76
	UDP glucose	0.004	0.001	0.003	0.001	0.003	0.001	0.003	0.001	0.76
	Xanthine	0.002	0.000	0.008	0.002	0.007	0.002	0.006	0.002	0.24
	Xylose	0.040	0.007	0.044	0.008	0.045	0.009	0.038	0.006	0.59
Energy metabolism	Acetic acid	2.865	0.248	3.065	0.323	2.848	0.202	2.790	0.258	0.78
	Fumaric acid	0.065	0.011	0.081	0.011	0.057	0.007	0.049	0.007	0.32
	Malic acid	0.060	0.008	0.073	0.014	0.059	0.008	0.060	0.010	0.77
	Butyric acid	0.351	0.040	0.409	0.037	0.38	0.028	0.353	0.035	0.76
	Propionic acid	0.731	0.082	1.059	0.151	1.068	0.108	0.792	0.085	0.83
	Succinic acid	0.026	0.003	0.036	0.007	0.032	0.009	0.041	0.012	0.86
	Valeric acid	0.819	0.094	0.862	0.097	0.904	0.087	0.861	0.099	0.92
Other	Glycerophosphorylcholine	0.008	0.002	0.010	0.002	0.012	0.004	0.006	0.001	0.24
	Nicotinate	0.024	0.003	0.029	0.004	0.032	0.004	0.026	0.003	0.24
Phenol metabolism	P-cresol	0.025	0.004	0.027	0.004	0.026	0.004	0.029	0.004	0.68

[1] q-values (FDR-adjusted p-values)

Supplementary Figure S1A

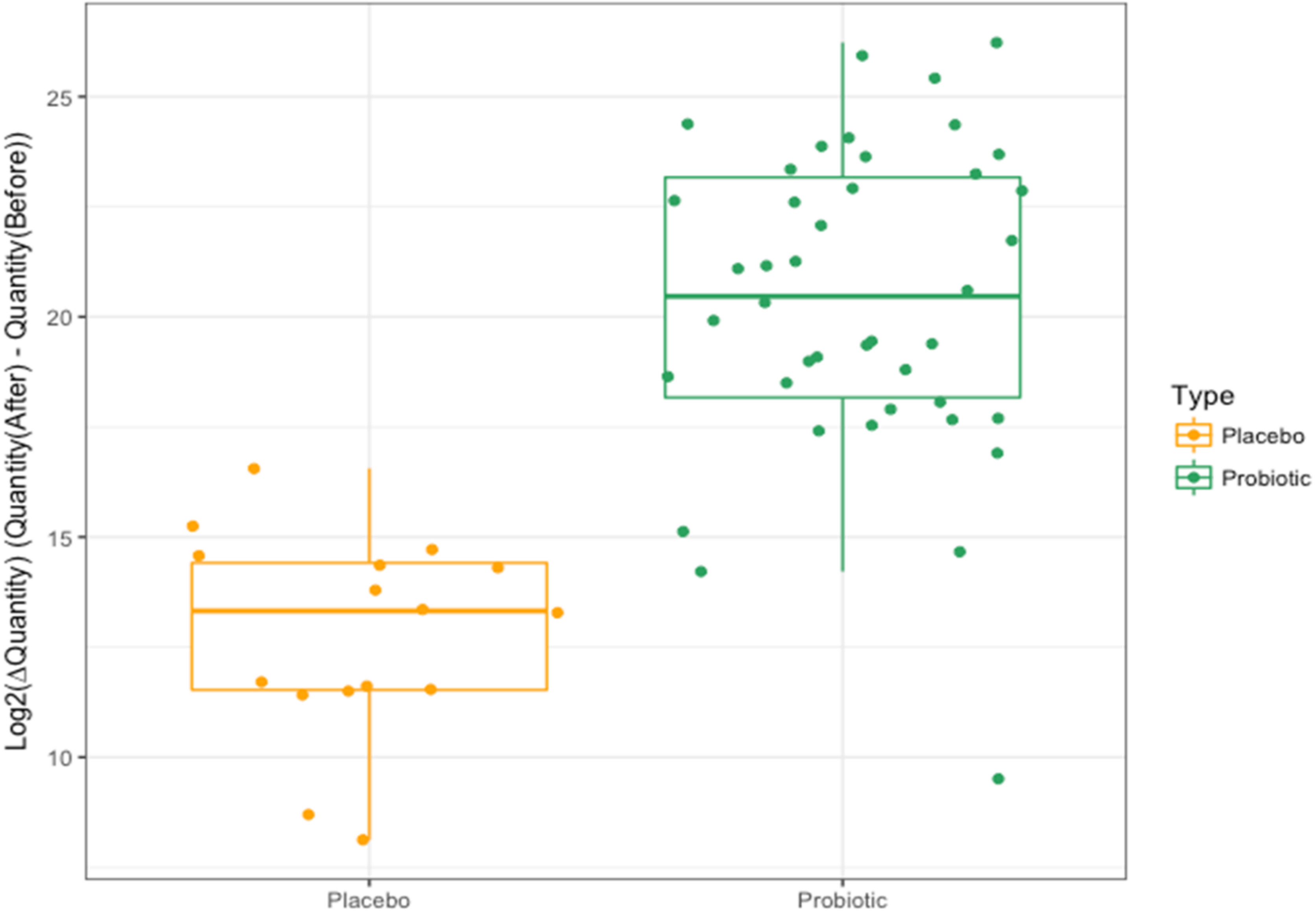

Supplementary Figure S1B

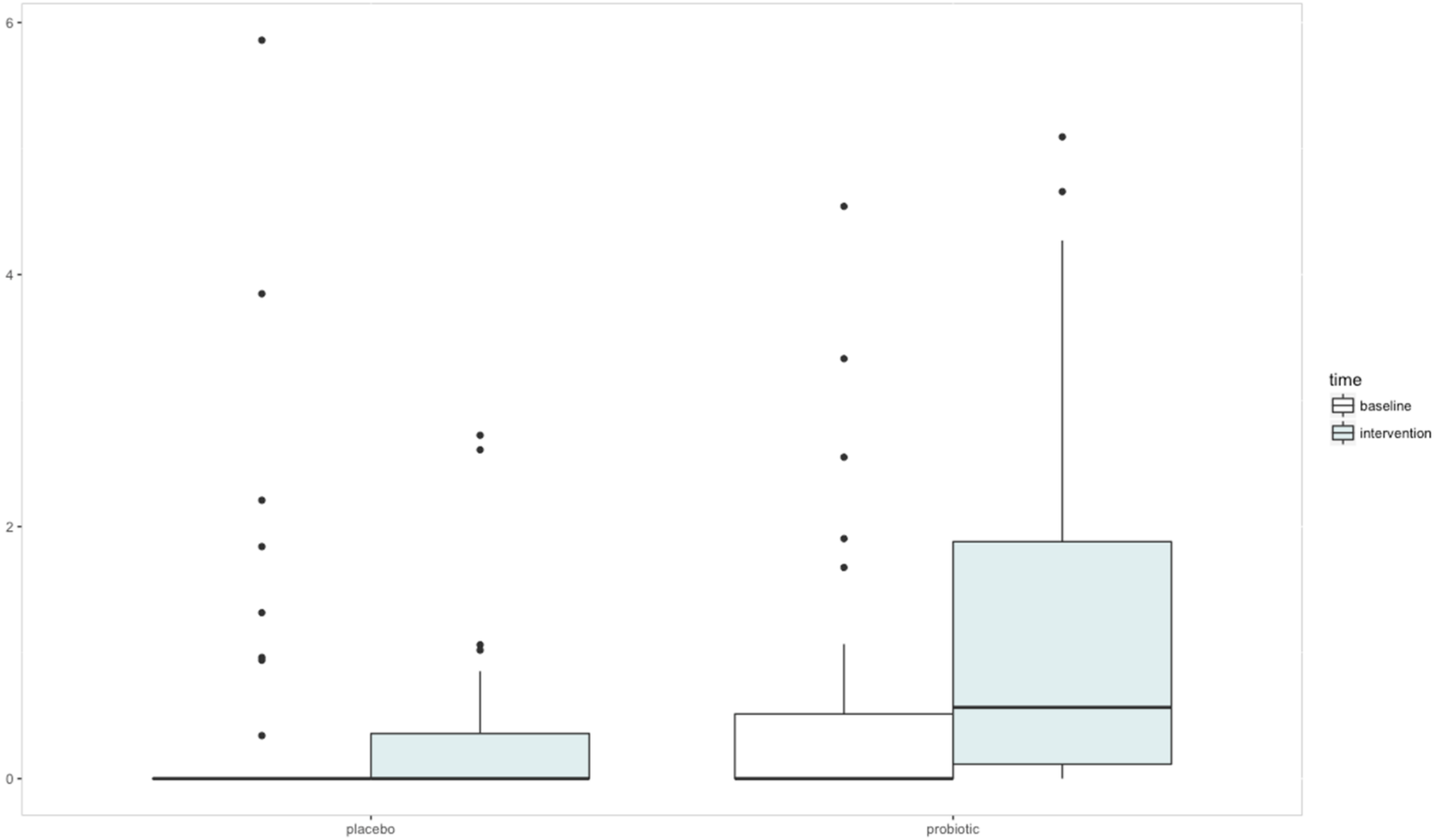

Supplementary Figure S2

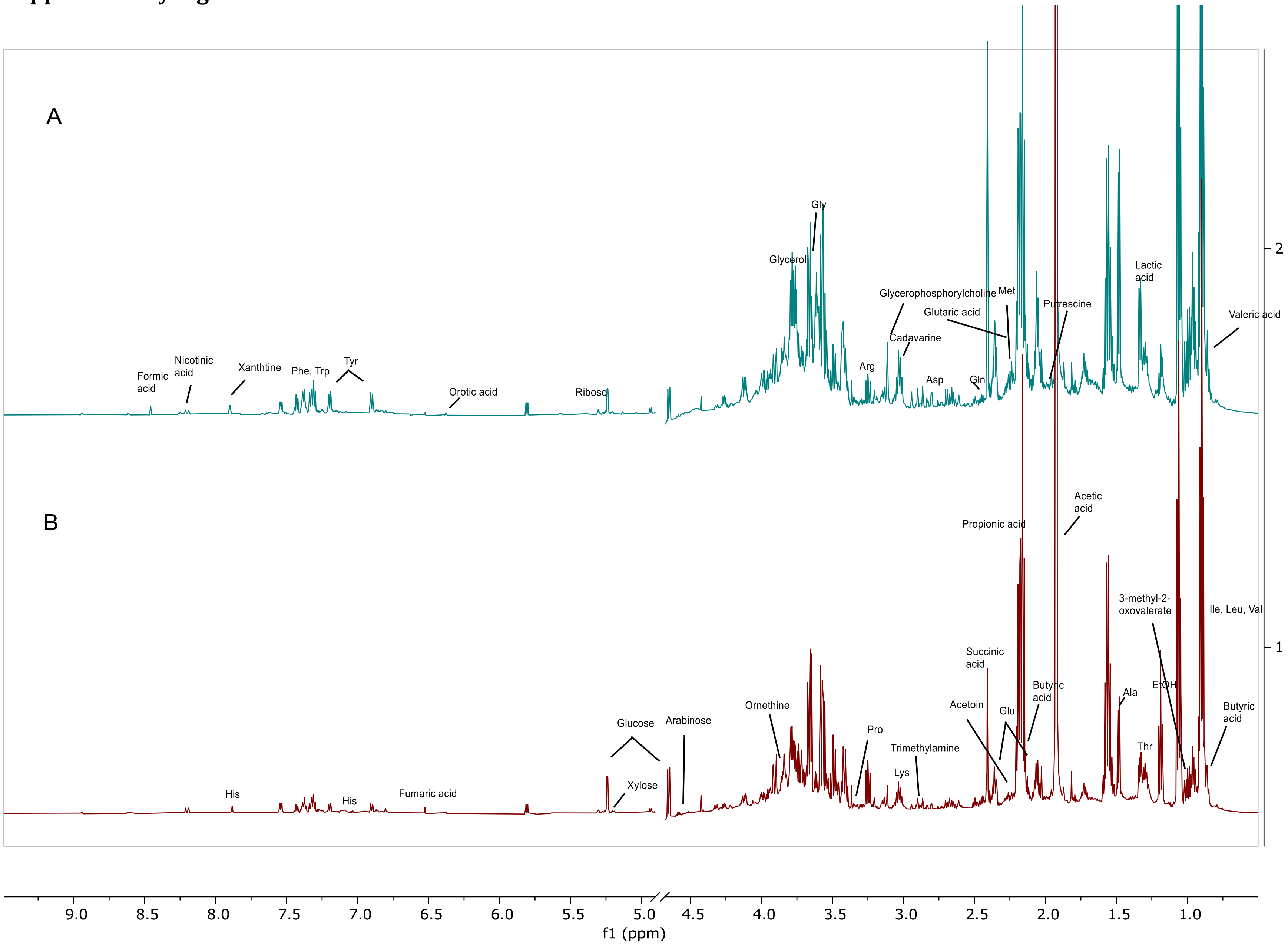

Supplement: Supplementary file 1 [file microorganisms-12-00796-s001.zip › microorganisms-2949072-supplementary.pdf]
